# Foreign Body Response to Intracortical Microelectrodes Is Not Altered with Dip-Coating of Polyethylene Glycol (PEG)

**DOI:** 10.3389/fnins.2017.00513

**Published:** 2017-09-14

**Authors:** Heui C. Lee, Janak Gaire, Seth W. Currlin, Matthew D. McDermott, Kinam Park, Kevin J. Otto

**Affiliations:** ^1^Weldon School of Biomedical Engineering, Purdue University West Lafayette, IN, United States; ^2^J. Crayton Pruitt Family Department of Biomedical Engineering, University of Florida Gainesville, FL, United States; ^3^Department of Neuroscience, University of Florida Gainesville, FL, United States; ^4^Department of Industrial and Physical Pharmacy, Purdue University West Lafayette, IN, United States; ^5^Department of Materials Science and Engineering, University of Florida Gainesville, FL, United States; ^6^Department of Neurology, University of Florida Gainesville, FL, United States; ^7^Nanoscience Institute for Medical and Engineering Technology, University of Florida Gainesville, FL, United States

**Keywords:** chronic neural implant, foreign body response (FBR), polyethylene glycol (PEG), CX3CR1-GFP, neuroprosthetics

## Abstract

Poly(ethylene glycol) (PEG) is a frequently used polymer for neural implants due to its biocompatible property. As a follow-up to our recent study that used PEG for stiffening flexible neural probes, we have evaluated the biological implications of using devices dip-coated with PEG for chronic neural implants. Mice (wild-type and CX3CR1-GFP) received bilateral implants within the sensorimotor cortex, one hemisphere with a PEG-coated probe and the other with a non-coated probe for 4 weeks. Quantitative analyses were performed using biomarkers for activated microglia/macrophages, astrocytes, blood-brain barrier leakage, and neuronal nuclei to determine the degree of foreign body response (FBR) resulting from the implanted microelectrodes. Despite its well-known acute anti-biofouling property, we observed that PEG-coated devices caused no significantly different FBR compared to non-coated controls at 4 weeks. A repetition using CX3CR1-GFP mice cohort showed similar results. Our histological findings suggest that there is no significant impact of acute delivery of PEG on the FBR in the long-term, and that temporary increase in the device footprint due to the coating of PEG also does not have a significant impact. Large variability seen within the same treatment group also implies that avoiding large superficial vasculature during implantation is not sufficient to minimize inter-animal variability.

## Introduction

Implantable neural probes hold the promise of providing functional recovery to individuals suffering from traumatic injuries or neurological disorders (Taylor et al., 2002; Hochberg et al., 2012). A major dilemma of using such devices is that their functionality degrades over time, which eventually leads to the inability of discriminating relevant neural signals from background noise (Liu et al., 1999; Vetter et al., 2004; Williams et al., 2007). In an attempt to resolve the biological aspects of this issue, researchers have modulated multiple factors including: device architecture/material type/flexibility (Seymour and Kipke, 2007; Karumbaiah et al., 2013; Xie et al., 2015; Lee et al., 2017a,b; Luan et al., 2017), bioactive coatings (Pierce et al., 2009; Azemi et al., 2011; Kozai et al., 2012a; Rao et al., 2012), and drug delivery schemes (Shain et al., 2003; Zhong and Bellamkonda, 2007). In parallel to these engineering mitigation strategies, there are ongoing attempts to discover the precise biotic and abiotic mechanisms of implant failure in order to develop strategies to improve the functional lifetime of neural implants (Barrese et al., 2013, 2016).

One of the most common and indirect methods for evaluating the effectiveness of these intervention strategies is looking at markers of the FBR. Typically, implanted microelectrodes in the brain instigate a neuro-inflammatory cascade that involves infiltration of plasma proteins, monocytes, macrophages, and leukocytes from breached blood-brain barrier (BBB) activation, and recruitment of microglia and macrophages to the injury site, activation of astrocytes to initiate astrogliosis, and a loss of neurons near the implant (Edell et al., 1992; Biran et al., 2005; Prasad et al., 2012; Potter-Baker et al., 2014; Jorfi et al., 2015). A severe FBR generally results in a large neuronal loss in the long-term (Prasad et al., 2012; Potter et al., 2013). Thus, reducing the FBR has been considered essential to achieve long-term functionality of neural implants.

A number of neural-interface strategies have used the biocompatible polymer poly(ethylene glycol) (PEG). Examples include using PEG as an adhesive to aid insertion (Gage et al., 2012), a stiffening agent for inserting flexible probes (Felix et al., 2013; Lecomte et al., 2015; Lee et al., 2017a), or as a vector for molecules to deter protein adsorption (Kozai et al., 2012a; Gutowski et al., 2014; Sommakia et al., 2014b). While the exact mechanisms are unknown, there are studies suggesting the use of PEG as a standalone treatment for spinal cord injury (SCI) (Luo et al., 2002; Estrada et al., 2014), traumatic brain injury (TBI) (Liu et al., 1989; Koob et al., 2005), and peripheral nerve damage (Britt et al., 2010). It was reported that PEG reduces oxidative stress and repairs damaged cell membranes which contribute to enhanced anatomical and functional recovery over a few hours (Liu et al., 1989; Luo et al., 2002; Koob et al., 2005) to weeks or months (Britt et al., 2010; Estrada et al., 2014). We have previously evaluated tissue responses to PEG-coated intracortical devices and found that PEG prevents glial cell adsorption but does not alter neuronal responses in the short-term in an *in vitro* setting (Sommakia et al., 2014a,b). However, the consequence of using PEG for brain-implanted microelectrodes in the chronic phase has not been investigated. Moreover, our recent study with flexible neural probes employed PEG as the stiffener for insertion (Lee et al., 2017a), and there remains a need to evaluate if any unforeseen effect of PEG has confounded the outcome of the study.

In this paper, we evaluated the brain's biological response to PEG-coated silicon probes and non-coated silicon probes at 4 weeks using two different mouse strains: wild-type (WT, C57BL/6) and CX3CR1-GFP (B6.129P-CX3CR1^−/−^). CX3CR1-GFP, a transgenic mouse model, which has its fractalkine receptor replaced with green fluorescent protein (GFP) (Jung et al., 2000), expresses GFP in immune cells including microglia and has been instrumental in studying microglial activity both *in vivo* and *ex vivo* in normal and injury models (Nimmerjahn et al., 2005; Kozai et al., 2012b, 2016; Lee et al., 2017a). However, the consequence of genetic manipulation in microglial cells on overall tissue response to neural implants remains unknown. We first conducted the PEG-coat vs. no-coat experiment on WT, and replicated on CX3CR1-GFP to identify that the observation from WT mice is consistent and if any difference exists between WT and CX3CR1-GFP.

## Materials and methods

### Probe preparation and PEG-coating

Planar “Michigan type” silicon microelectrodes, 249 μm wide and 15 μm thick, were used in this study (GP_1x16_249, NeuroNexus, Ann Arbor, MI; same dimension used in Lee et al., 2017a,b). Probes were “raw,” meaning that no electrical backbone was attached to efficiently conduct histological assessment. Four kilodaltons PEG was chosen for its superior anti-biofouling property (Su et al., 2009). Probes and PEG (4 kDa MW, Sigma, St. Louis, MO) were autoclaved for 30 min at 120°C 1 day prior to surgery. In an aseptic setting, PEG was melted on a hot plate at 80°C and the probes were dip coated in PEG for 5 s. After allowing the probes to air dry they were sealed in a sterilized container until surgery.

**Figure 1:**
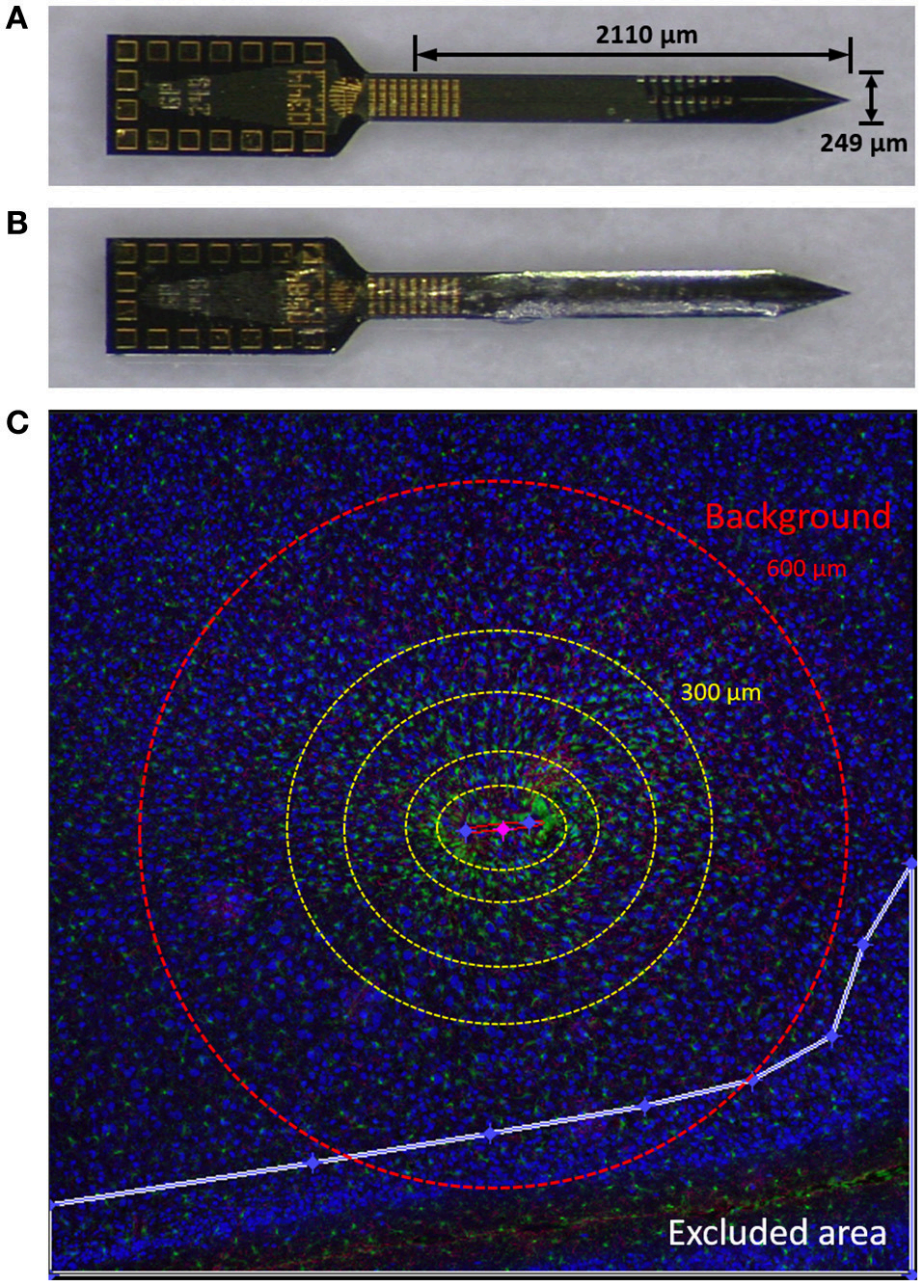
Materials and methods. **(A)** Planar silicon probe GP_1x16_249 used in this study. **(B)** Probe dip-coated with PEG. **(C)** An image from a typical tissue slice demonstrating using Minute v1.5 for quantitative analysis of immunolabels. Yellow dotted concentric circles denote binned interval (0–50, 50–100, 100–200, and 200–300 μm from the defined probe contour). GFP (green), GFAP (red), and NeuN (blue) are shown. Outside the red dashed circle (600 μm) was considered background. The white line denotes the border of an exclusion area in this image. NeuN is pseudo-colored blue in this figure and yellow for subsequent figures.

A separate set of probes coated with PEG were taken for profilometry thickness measurements (Alpha Step 500, Tencor, Milipitas, CA). The maximum thickness of the PEG-coatings was 46.05 μm (±4.86 μm, s.e.m., *N* = 9) on one side of the broad surface. Figures 1A,B show pictures of non-coated and PEG-coated probes.

### Surgical procedures

All surgeries and animal experiments were performed in accordance with the University of Florida Institutional Animal Care and Use Committee (IACUC) guidelines.

Procedures are almost identical to our previous study (Lee et al., 2017a). Briefly, 2–3 months old (18–28 g) WT mice (C57BL/6, *N* = 4) and CX3CR1-GFP mice (B6.129P-CX3CR1^−/−^, Stock #005582, The Jackson Laboratory, Bar Harbor, ME, *N* = 4) were bilaterally implanted with a PEG-coated device in one hemisphere and a non-coated device in the other hemisphere. Mice were anesthetized with isoflurane (3% induction, 1.5% maintenance) and kept warm using a heating pad. Vital signs were monitored throughout the surgery with a pulse oximeter (MouseOx, Kent Scientific, Torrington, CT). After a small portion of scalp was removed, a craniotomy above each brain hemisphere was performed using a microdrill. A probe was mounted onto a piezoelectric actuator (PiLine M-663, Physik Instruments, Karlsruhe, Germany) and inserted into one hemisphere 1.2 mm from the cortical surface at 100 mm/s. Sensorimotor cortical regions (~1 mm posterior and 1.5 mm lateral to bregma) were targeted, however small deviations occurred to avoid large surface vasculature. This procedure was then repeated on the contralateral hemisphere. After the insertion in both hemispheres, craniotomies were covered with silicone elastomer (Kwik-Sil, WPI, Sarasota, FL) followed by dental acrylic (Fusio Liquid Dentin, Pentron, Orange, CA) to secure the devices. Post-operative care involved applying antibiotic ointment (Actavis, NC) around the cut areas and maintaining the body temperature. Meloxicam (Norbrook, United Kingdom) was administered pre- and up to 3 days post-surgery to minimize discomfort.

### Tissue preparation

After 4 weeks of implantation, mice were deeply anesthetized with 5% isoflurane and transcardially perfused with 20 mL of ice cold phosphate buffered saline (PBS, pH 7.4) followed by 20 mL of ice cold 4% paraformaldehyde (PFA, pH 7.4) solution. Heads were then isolated and soaked in 4% PFA for 24 h at 4°C for post-fixation. After rinsing with PBS, brains were carefully extracted from the head and cryopreserved in 30% sucrose in PBS for 24–48 h at 4°C. Brain tissues were flash frozen in 2-methyl butane at −40°C for 2 min and equilibrated to −20°C before cryosectioning. Tissues were lightly embedded with Optimal Cutting Temperature (OCT) medium (Sakura Finetek, Alphen aan den Rijn, The Netherlands) then horizontally sectioned into 25 μm slices and transferred to electrostatic adhesive glass slides (Superfrost Plus, Thermo Fisher Scientific, Waltham, MA). Harvested slices were kept at 4°C for no longer than 1 week before immunolabeling.

### Immunohistological processing

Tissue slices were allowed to sit at room temperature (RT) for 30 min for secure adhesion onto slides. Tissue slices were rinsed three times with PBS for 5 min each to remove residual OCT and then blocked in blocking buffer (4% goat serum, 0.3% Triton-X in PBS) for 2 h at RT. Primary antibodies diluted in blocking buffer were applied and incubated for 20–24 h at 4°C. Following primary incubation, tissue slices were washed five times with PBS for 5 min each to remove any unbound primaries. Corresponding secondary antibodies diluted in blocking buffer were then applied to the tissue slices and incubated for 2 h at RT. After 5 min of five subsequent washes with PBS, tissue slides were cover slipped using VectaShield mounting medium (H-1000, Vector Lab, Burlingame, CA).

Tissue slices were prepared in two sets that alternate every 25 μm in depth. The first set of slices were stained with CD68 and IgG. The second set of slices were stained with GFAP and NeuN. Note that for assessing BBB leakiness, the secondary antibody (i.e., anti-mouse IgG 555) was directly applied to the tissue without a primary. Antibodies used in this study are listed in Table 1.

**Table 1:** List of primary and secondary antibodies.

**Primary antibodies**	**Secondary antibodies (host: goat)**	**Target of interest**
Rat anti-CD68 (1:250, ab53444, Abcam)	Anti-rat IgG 647 (1:500, ab150159, Abcam)	Activated microglia/macrophages
Secondary serves as primary	Anti-mouse IgG 555 (1:250, ab150114, Abcam)	Blood-brain barrier leakiness
Chicken anti-GFAP (1:500, AB5541, Millipore)	Anti-chicken IgG 555 (1:500, A21437, Life Technologies)	Astrocytes
Rabbit anti-NeuN (1:250, MABN140, Millipore)	Anti-rabbit IgG 633 (1:500, A21071, Life Technologies)	Neuronal nuclei

### Imaging and quantitative analysis

Fluorescence images were taken with a Zeiss LSM 710 laser scanning confocal microscope (Carl Zeiss, Jena, Germany). A 2 × 2 tile was captured using a 10X objective to obtain a wider view of the device-tissue interface for quantitative analysis. To minimize the depth dependent variability (Woolley et al., 2013), two slices per sample, roughly from 450 to 600 μm down the cortical column, were used for quantitative analysis. A 20X objective was used for representative qualitative figures. A maximum intensity projection was used for 20X stacks to span 10 μm. Qualitative figures presented in this paper were contrast enhanced and pseudo-colored for visual clarification.

We used Minute v1.5 (Potter et al., 2012; Potter-Baker et al., 2014) for the quantitative analysis of immunolabels. Briefly, an ellipse was drawn to define the contour of the device track. Exclusion areas were drawn to limit the analysis to be conducted only on cortical areas of the device-containing hemisphere. For each 5 μm concentric ring, expanding from the defined ellipse, the average label intensity and the area of the ring was calculated. The strength of Minute v1.5 is that it allows us to utilize the entirety of an image, which is useful for reducing potential bias from using only a portion of the image for quantification. An illustration is depicted in Figure 1C.

We binned in intervals: 0–50, 50–100, 100–200, 200–300, and 300–500 μm. Average intensities of 5 μm rings were weighted by each area over the total area and summed to generate an average intensity of each binned interval (Potter et al., 2012; Potter-Baker et al., 2014). Each average binned interval was normalized to background, taken as the ring 600–800 μm away from the device contour. Neuronal nuclei (NeuN) were counted with ImageJ (Schneider et al., 2012) using a custom, automated cell counting script.

### Statistical analysis

Statistical analyses were performed using Prism 7.00 statistical analysis software (GraphPad Software, La Jolla, CA). A two-way ANOVA was performed taking coating scheme (PEG vs. No-Coat) and binned interval as the two independent variables. Then, we blocked each animal to eliminate inter-animal variability and ran paired *t*-tests to directly compare, within each interval, a PEG-coated device implanted hemisphere with its contralateral non-coated device implanted hemisphere. Tests were run on WT and CX3CR1 independently to verify the results' consistency.

## Results

### Microglia/macrophage characteristics

**Figure 2:**
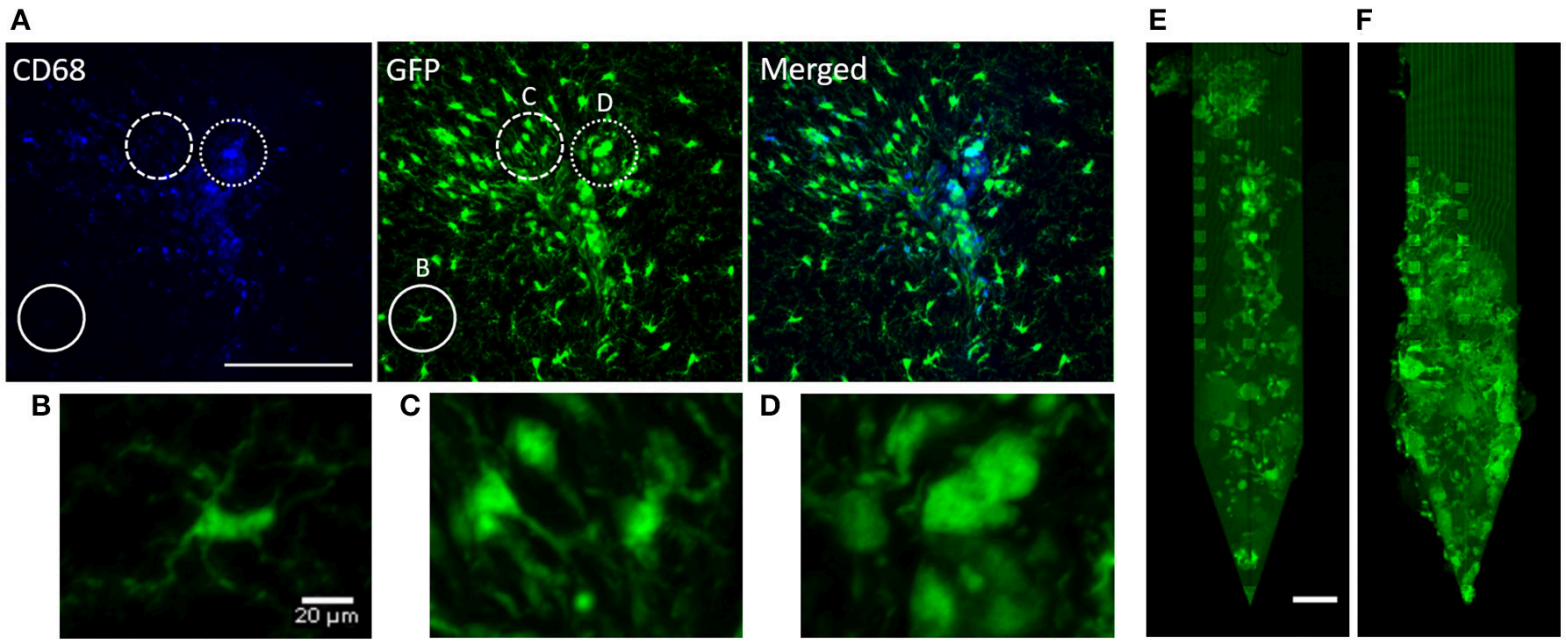
Qualitative demonstration of microglial response. **(A)** CD68 fluorescence, autofluorescent GFP, and a merged image of a CX3CR1-GFP non-coated device-tissue interface at 4 weeks. The solid circle highlights non-activated, ramified microglia that do not co-localize with CD68 (high-magnification image shown in **B**). The dashed circle highlights activated microglia that are extending their processes toward the implant (high-magnification image shown in **C**). The dotted circle highlights highly activated microglia/macrophages that look to be lumped together (high-magnification image shown in **D**). Autofluorescent GFP of **(E)** an extracted PEG-coated probe and **(F)** a non-coated probe. Scale bars in **(A)** and **(E)** are 100 μm and **(B)** is 20 μm.

To identify whether observed immune responses correspond with previously published work (Biran et al., 2005; Kozai et al., 2012b) we took high magnification images of microglia in the proximity of the implant to examine their morphology. Figure 2A shows CD68, GFP, and merged images of a device-tissue interface taken with a 20X objective. It can be seen in Figures 2A–D that activated microglia/macrophages extended their processes toward the implant track (Figure 2C) and/or fused together making them harder to detect as individual cells (Figure 2D). They looked largely different from ramified microglia that are in a resting state (Figure 2B). This microglial activity was also identified by co-localization of GFP with CD68 immunoreactivity. Of the microglia co-localized with CD68 near the implant track, no particular morphological differences were noted between PEG-coat and no-coat groups (data not shown). Figures 2E,F are examples of microglial adsorption to PEG-coated and non-coated devices, respectively. It is intuitive and supported by previous studies that PEG prevents glial cell adsorption to the device in the short-term, but here only few devices were retained intact and no statistical conclusion could be drawn at 4 weeks. It appeared that microglia did not preferentially adhere to a specific region of the device such as electrode sites.

### Quantitative analysis of immunolabels

There was no significant interaction effect between the coating and animal strain (*p* > 0.05). Hence we mainly compared the effect of PEG coating with WT mice, and then repeated with CX3CR1-GFP mice.

**Figure 3:**
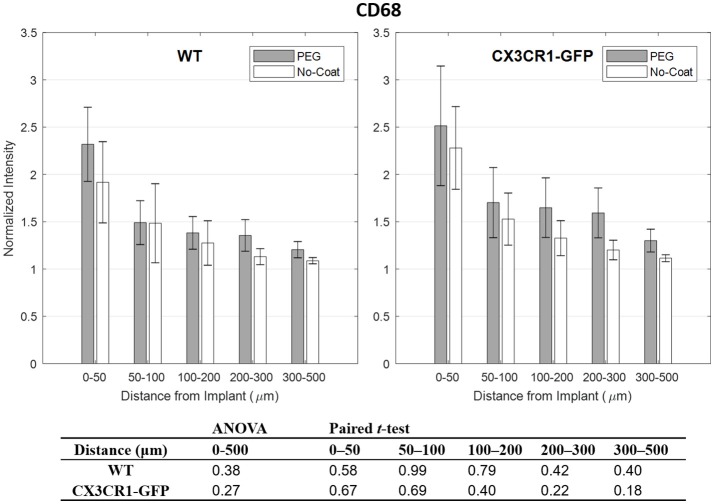
Normalized fluorescence intensity of CD68 as a function of distance from the implant at 4 weeks. *p*-values of the two-way ANOVA for the bins within 0–500 μm interval and paired *t*-tests for each binned interval are presented in the table. Error bars denote standard error mean (*N* = 4).

Quantitative analysis of normalized CD68 fluorescence intensity *vs*. distance from the implant is depicted in Figure 3. For both WT and CX3CR1-GFP mice, CD68 was strongest in the 0–50 μm interval and gradually decreased to the background level in the outer regions. No statistical significance was found between PEG-coat vs. no-coat with the ANOVA test (*p* > 0.05). Paired *t*-tests also did not show statistical difference in any of the binned intervals (*p* > 0.05). Although statistically not significant, PEG-coated devices generally had higher intensity of CD68 than non-coated devices.

**Figure 4:**
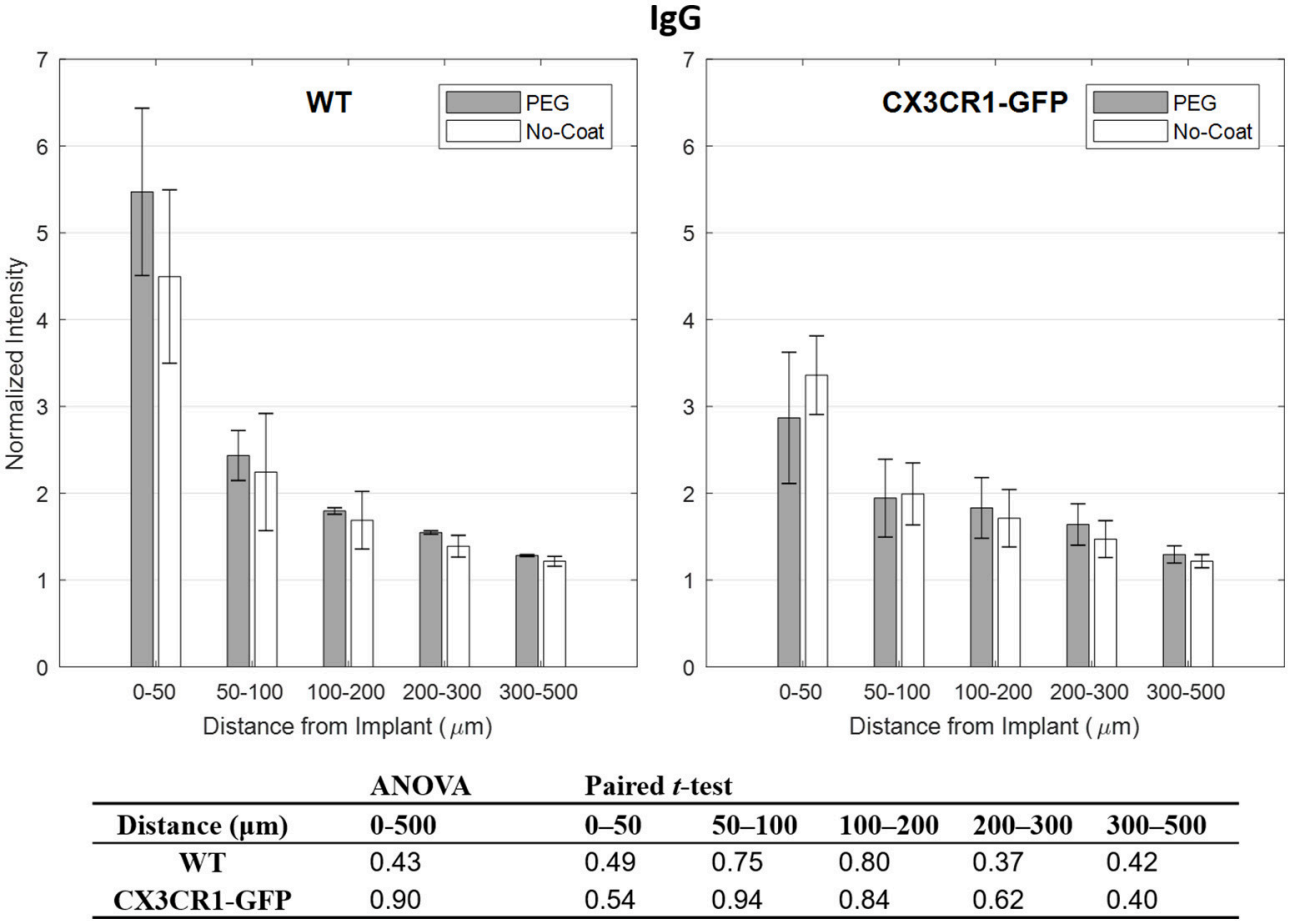
Normalized fluorescence intensity of IgG as a function of distance from the implant at 4 weeks. *p*-values of the two-way ANOVA for the bins within 0–500 μm interval and paired *t*-tests for each binned interval are presented in the table. Error bars denote standard error mean (*N* = 4).

IgG is known to correlate well with markers for activated microglia, such as CD68, as it is indicative of chronic BBB leakage which is largely affected by the perturbation of BBB from inflammatory molecules released by activated glial cells (Potter et al., 2013; Saxena et al., 2013). However, it has also been shown that IgG can be highly fluctuating due to its dependency to nearby vasculature (Lee et al., 2017a). In Figure 4 we see that IgG subsides to the background level, but in the close proximity to the implant the intensity is highly variable. We also see the same trend that is seen from CD68 that PEG-coated devices show higher IgG level than the non-coated devices, except for the bins 50–100 and 50–100 μm of CX3CR1-GFP mice. No statistical significance between PEG-coat and no-coat was found with both WT and CX3CR1-GFP mice in any of the binned intervals (*p* > 0.05).

**Figure 5:**
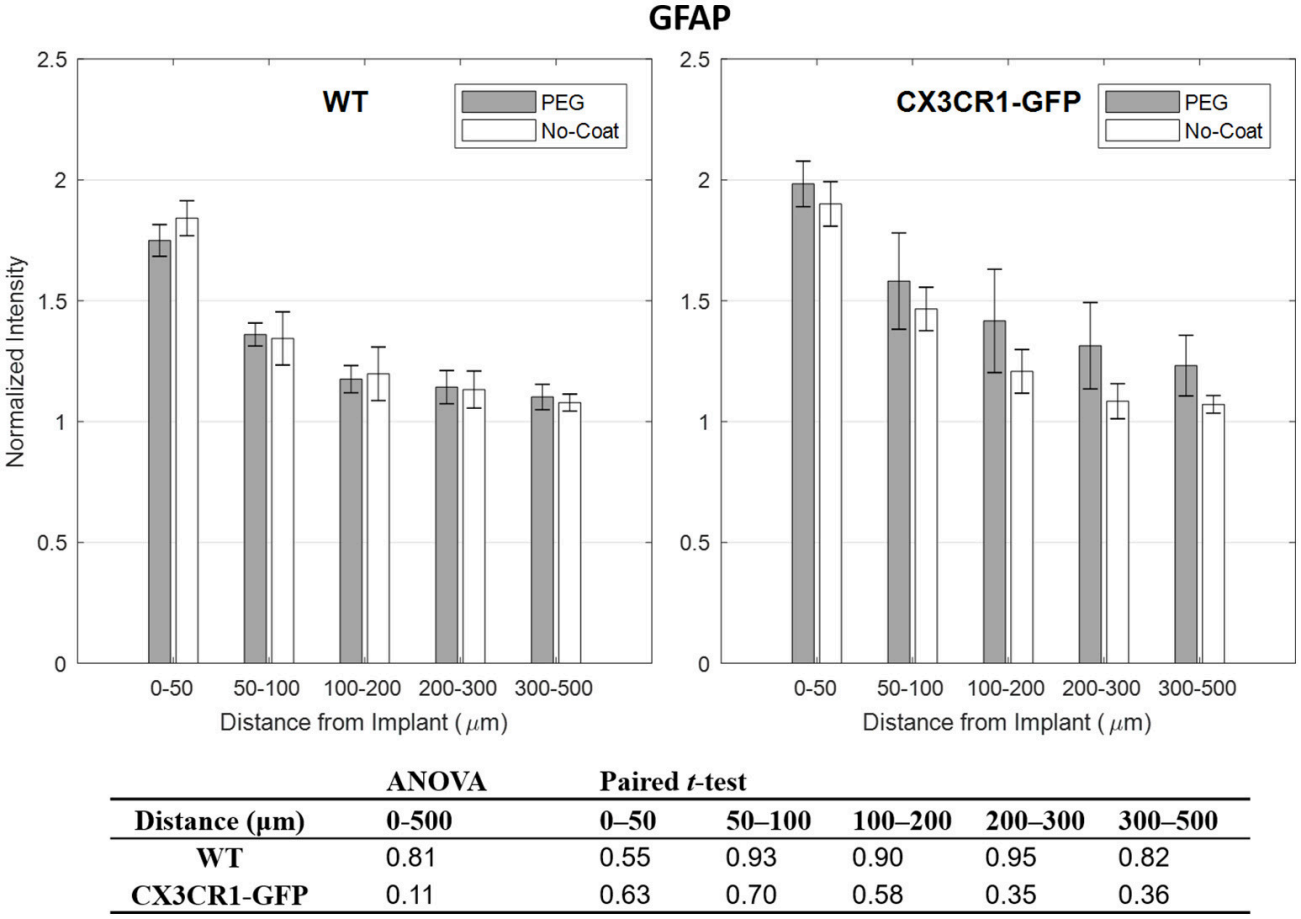
Normalized fluorescence intensity of GFAP as a function of distance from the implant at 4 weeks. *p*-values of the two-way ANOVA for the bins within 0–500 μm interval and paired *t*-tests for each binned interval are presented in the table. Error bars denote standard error mean (*N* = 4).

**Figure 6:**
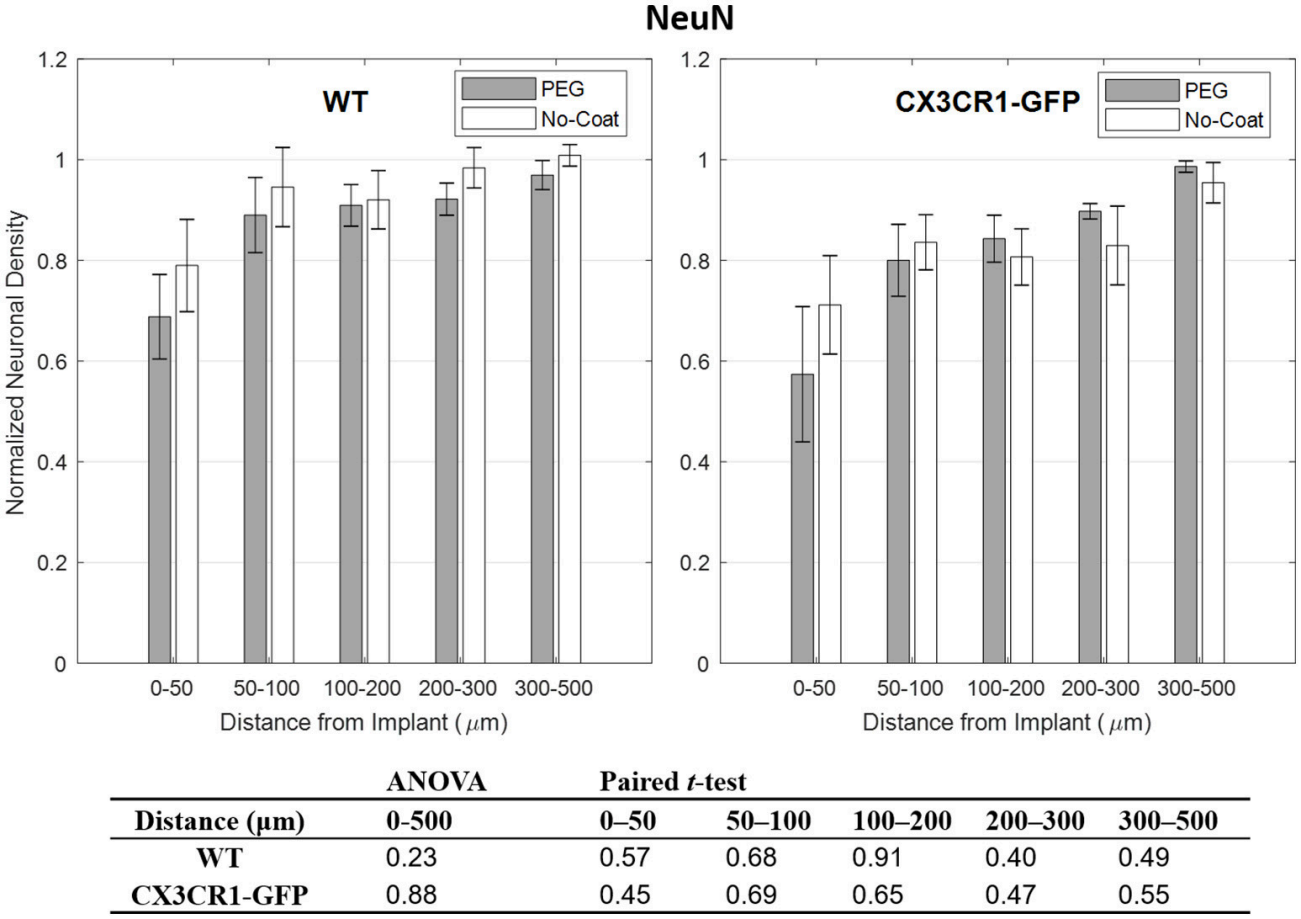
Normalized fluorescence intensity of NeuN as a function of distance from the implant at 4 weeks. *p*-values of the two-way ANOVA for the bins within 0–500 μm interval and paired *t*-tests for each binned interval are presented in the table. Error bars denote standard error mean (*N* = 4).

Compared to other immunolabels used in this study, the mean values of GFAP were stable especially at 0–50 μm indicated by relatively small error bars. However, no statistical difference in normalized GFAP fluorescence intensity was found between PEG-coat vs. no-coat with the two statistical tests as seen in Figure 5. The statistical non-significance was also retained in neuronal density as seen in Figure 6. NeuN counts were more variable in the 0–50 μm bin but gradually became stable at distant intervals. Similar to the trend seen from CD68 and IgG, the mean neuronal densities of PEG-coat were lower than that of no-coat without statistical significance. The background neuronal densities were 2347 (±94, s.e.m., *N* = 8) cells/mm^2^ for GFP mice and 2376 (±53, s.e.m., *N* = 8) cells/mm^2^ for WT mice, which are in the range of previously reported neuronal densities in healthy mouse cortex (Schuz and Palm, 1989).

### Variability of FBR

**Figure 7:**
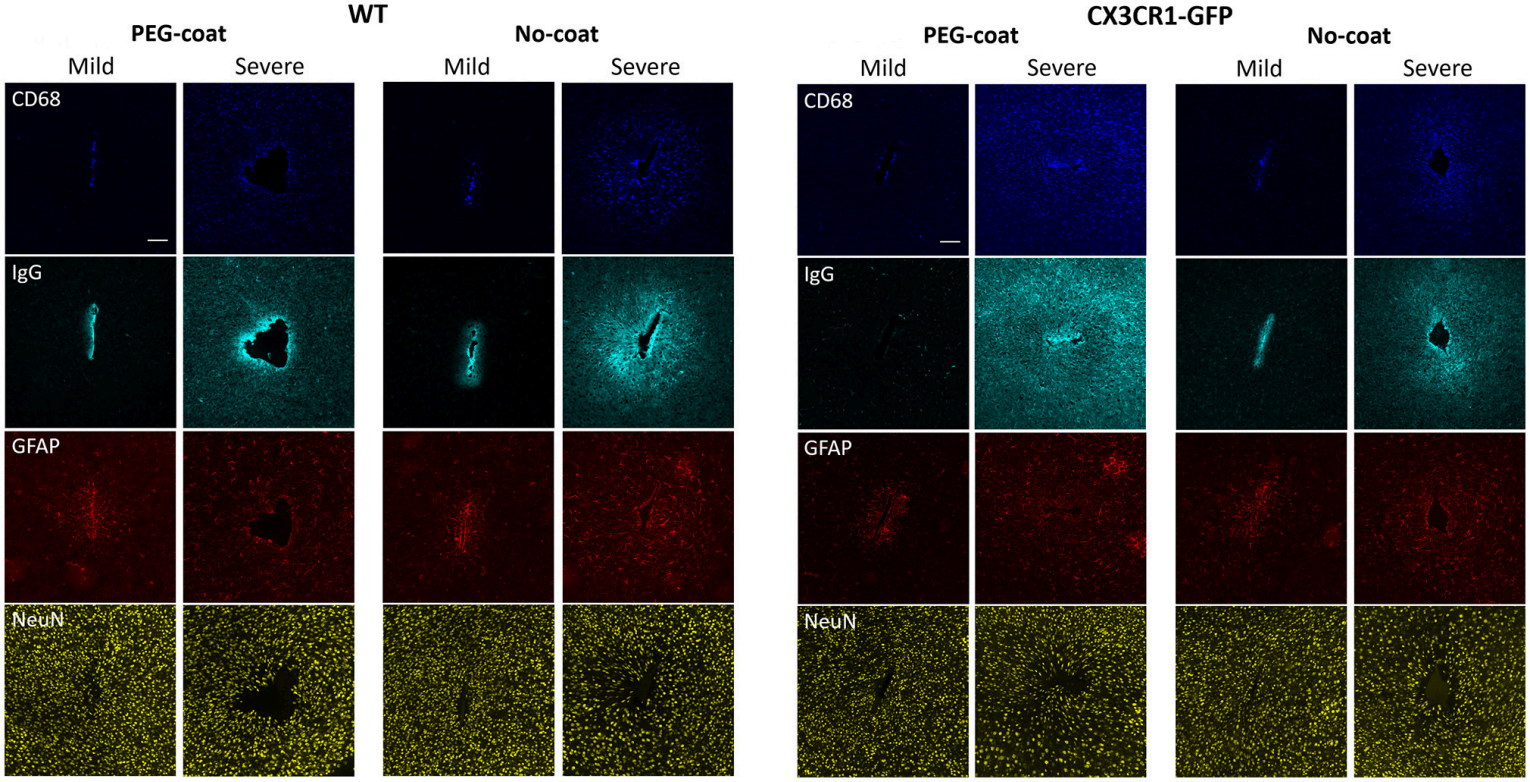
Representative images of CD68, IgG, GFAP, and NeuN immunolabels in WT mice and CX3CR1-GFP mice that show either a mild or a severe FBR. Images in the same column are from the same animal. Scale bars are 100 μm.

Even within the same treatment group vastly different tissue responses were observed. PEG-coated or non-coated devices, regardless of implanted in CX3CR1-GFP mice or WT mice, caused either a mild response or a severe response, as can be seen in Figure 7. In mild responses, CD68, IgG, and GFAP fluorescence intensities were relatively weaker and only localized to the implant track. In severe responses, the fluorescence intensities were stronger and largely diffused to the outer intervals. Mild responses were also manifested by a relatively higher neuronal density near the implant track than that of severe responses. When there was a large implant track, it always accompanied a severe response. However, a severe response did not always indicate that its implant track was large (e.g., in Figure 7 CX3CR1-GFP PEG-coat severe response).

## Discussion

### Microglial response

As described in a number of reports, activated microglia play a key role in the neuro-inflammatory response to foreign objects by secreting various pro-inflammatory and cytotoxic factors which propagate the neuro-inflammatory cascade (Nimmerjahn et al., 2005; Kozai et al., 2012b). We see in Figures 2A–D that our samples reflect morphologic changes upon activation that have been well-established in this field. Kozai et al. showed that microglia, presumably surveying the local environment, extend their processes toward the foreign object at the onset of the phagocytic state (Kozai et al., 2012b). Microglia then migrate toward the device surface and create a microglial sheath. We saw the extension of processes at 4 weeks post-implantation, indicating that there was ongoing inflammation near the implant track. The GFP in Figure 2D, which highly co-localizes with CD68 activity, indicated maximally activated microglia or macrophages. This is supported by studies showing a large microglial response near the implant in the early chronic phase and a subsided response later in the chronic phase.

Previous acute studies have revealed that PEG prevents protein adsorption to silicon or polymer based probes by creating a hydrophilic layer in between the device-tissue interface (Gutowski et al., 2014; Sommakia et al., 2014a,b). An *in vitro* study by Gutowski et al. showed that microglia are seldom found on PEG-decorated devices at 24 h post-implantation (Gutowski et al., 2014). Another *in vitro* study by Sommakia et al. showed that a significant difference in microglial population exists between PEG-coated and non-coated devices up to 50 μm away at 7 days, although the difference was not as dramatic as that by Gutowski et al. at 24 h (Sommakia et al., 2014b). In this study, it appears that fewer microglia are attached to the surface of the device at 4 weeks with qualitative examination (Figures 2E,F), but the intensity profiles around the device suggest that there was no significant difference (Figure 3). The difference in results is mainly due to the longer implantation time, but other factors also could have played such as the *in vivo* environment that accelerates removal of the PEG layer surrounding the device and/or scraping off of the PEG layer during the insertion process. By 4 weeks, the 4 k PEG layer would have long been gone although the dissolution and degradation time may vary depending on the molecular weight and the surrounding medium (Glastrup, 1996). Note that our work herein focuses on the impact of acute delivery of PEG, which presumably alters the onset of inflammatory cascade, on the chronic FBR.

### Impact of PEG-coating

Conflicting expectations may exist regarding the use of PEG coatings. It could be that (1) the ameliorative effect of PEG reduces the onset of the FBR and subsequently the chronic FBR, or (2) larger mechanical damage caused by the thick layer of coating exacerbate the chronic FBR.

PEG has been shown to be biocompatible and provide a beneficial effect on traumatic injury sites. PEG is not readily degraded upon hydration and interacts with the surrounding tissues until it dissolves away (Glastrup, 1996). Studies have reported that PEG reconstitutes damaged cell membranes which in turn promotes axonal regeneration in injured spinal cords (Luo et al., 2002; Estrada et al., 2014) or reduces traumatic or ischemic cell loss in the brain (Liu et al., 1989; Koob et al., 2005). Moreover, acute *in vivo/in vitro* tissue responses (Gutowski et al., 2014; Sommakia et al., 2014b) and impedance measurements (Sommakia et al., 2014a) suggest that PEG possesses anti-biofouling properties which prevent glial cell adsorption. The hydrated PEG layer can also work as a short term diffusion barrier. Despite the reported benefits of PEG, no significant difference in FBR was found between PEG-coat vs. no-coat cohorts in this study. A subtle difference existed in that PEG-coated devices had greater FBR than non-coated devices, especially in the 0–100 μm range. However, this difference was not statistically significant. Since PEG is presumed to be gone long before 4 weeks, and with no significant difference in FBR at 4 weeks, we expect little deviation from this trend beyond 4 weeks.

It is unclear whether PEG has little effect on reducing the FBR, or the beneficial effects of PEG were leveled off by a larger initial trauma caused by the thick coating. The increase in thickness of the penetrating profile was from 15 μm to ~100 μm, which could be sufficiently large to induce a difference when the thickness is retained throughout the implantation period (Karumbaiah et al., 2013). In our study, however, the thickness of the residing material was kept the same as control since the PEG layer dissolves away upon contact with the blood and cerebrospinal fluid. Skousen et al. reported that the surface area of the residing material is a significant factor when penetrating profiles are kept similar (Skousen et al., 2011). This also supports the idea that the dimension of the residing material is more impactful.

### Implications from CX3CR1-GFP mice

For CX3CR1-GFP mice, the involvement of CX3CR1 gene in accelerating or decelerating the secretion of neurotoxic factors in microglia is controversial. CX3CR1, a receptor present in microglia, interacts with the ligand fractalkine in neurons which is considered a mechanism of neuron-glia crosstalk. This poses the question of whether the deletion of CX3CR1 has a prominent impact on the various cells including neurons. Jung et al. found that the absence of CX3CR1 in microglia did not alter the microglial response to various inflammation models, one being a peripheral nerve axotomy (Jung et al., 2000). However, contradictory results were reported that CX3CR1 deletion may have a negative or positive effect on neurons. Cardona et al. demonstrated that CX3CR1 deficient mice either stimulated with lipopolysaccharide (LPS), given neurotoxins to induce Parkinson's disease symptoms, or genetically modified to induce amyotrophic lateral sclerosis resulted in more neurodegeneration than controls (Cardona et al., 2006). By contrast, Denes et al. and Donnell et al. reported that the lack of CX3CR1 had a neuroprotective effect in brain ischemia and spinal cord injury models, respectively (Denes et al., 2008; Donnelly et al., 2011). Among these, our results corresponded well with Jung et al. in which there was no significant impact with CX3CR1 deletion. Statistical tests comparing WT vs. CX3CR1-GFP showed no significant differences with any of the immunolabels (data not shown). It may be that there is no general rule for CX3CR1 deficient microglia to react to the induced inflammation, but rather the response largely depends on the type of stimuli and the target region of the body. The trend of PEG-coat vs. no-coat in WT mice was replicated with very similar characteristics, strengthening the idea that dip-coating of PEG has little impact on the chronic FBR.

### On the variability of the quantitative histology

Even if PEG-coating and/or the CX3CR1-GFP mouse model caused small differences in the FBR, the differences were not prominent compared to the innate variability of the neural implant with the current technical standard. The qualitative images in Figure 7 are indicative of the highly variable nature of the FBR to implants. Even though all the surgical and care plans remained the same, there was a large difference in the tissue response within the same group. This is in line with the discrepancies observed between studies or even within studies (Jorfi et al., 2015). Rousche et al. and Williams et al. showed that a large variability existed even between different shanks within a multi-shank device (Rousche and Normann, 1998; Williams et al., 2007). Our previous study with flexible neural probes also indicate that reducing the variability would enable accurate identification of critical factors responsible for chronic device failure (Lee et al., 2017a). Vascular damage is a probable suspect for this variability, as devices that sever large vasculature are reported to cause severe neuro-inflammation (Skousen et al., 2011; Saxena et al., 2013). Although surface vasculature can be avoided during implantation, vasculature that underlie brain parenchyma are hard to avoid unless identified. Emerging technologies such as 3D mapping of the brain prior to insertion may minimize BBB damage (Kozai et al., 2010). Although the breach of the BBB is inevitable, minimizing this variability will be critical in facilitating quantitative research.

## Conclusion

We have evaluated the FBR of implanted microelectrodes with and without PEG-coating in two mouse strains (WT and CX3CR1-GFP). Statistical analyses suggest that dip-coating of PEG does not result in a significant decrease or increase in the FBR at 4 weeks post-implant. This finding is supported by the consistency observed in the transgenic CX3CR1-GFP mouse model. This study confirms that an acute delivery of PEG does not result in significant mitigation of the chronic FBR. Comparison studies that utilize PEG for various purposes can be assured that PEG does not confound the study that assesses the degree of FBR in the long-term.

## Author contributions

HL has led the entire experiments and composing of the manuscript. JG assisted with surgeries and provided his insight to the research. SC helped with immunohistology. MM helped with profilometry. KP and KO funded and mentored the research. All authors participated in writing and revising of the manuscript.

### Conflict of interest statement

The authors declare that the research was conducted in the absence of any commercial or financial relationships that could be construed as a potential conflict of interest.
